# Agreement between corneal diameter measurements obtained with an optical biometer and a Placido-based topographer

**DOI:** 10.5935/0004-2749.2021-0325

**Published:** 2023

**Authors:** Eloisa Gomes Rosario M. Teixeira, Beatriz Fiuza Gomes, João Dominice Santana, Marcony R Santhiago, Adroaldo Alencar Costa, Haroldo V Moraes Jr

**Affiliations:** 1 Department of Ophthalmology, Hospital Federal de Bonsucesso, Rio de Janeiro, RJ, Brazil; 2 Department of Ophthalmology, Universidade Federal do Rio de Janeiro, Rio de Janeiro, RJ, Brazil; 3 Department of Ophthalmology, Universidade de São Paulo, São Paulo, SP, Brazil

**Keywords:** Corneal topography, Axial length, eye, Diagnostic techniques, Ophthalmological, Ophthalmological surgical procedures, Topografia da córnea, Comprimento axial do olho, Técnicas de diagnóstico oftalmológico, Procedimentos cirúrgicos oftalmológicos

## Abstract

**Purpose:**

The purpose of this study was to compare the white-to-white distance measurements of two devices (IOL Master 500 and Atlas corneal topographer) commonly used in clinical practice to determine if they were interchangeable. Providing information on instrument interchangeability could eliminate several unnecessary tests and consequently reduce the economic burden for the patient and society.

**Methods:**

In this prospective, comparative case series, the white-to-white distance was measured by independent examiners using the Atlas topographer (Carl Zeiss Meditec) and the IOL Master 500 (Carl Zeiss Meditec). One eye each of 184 patients was tested. Statistical analyses were performed using a paired *t*-test, Pearson correlation analysis, and Bland-Altman analysis to compare the measurement methods.

**Results:**

The mean white-to-white distance measurements with the Atlas topographer and the IOL Master 500 were 12.20 ± 0.44 mm and 12.12 ± 0.41 mm, respectively (p<0.001). The mean white-to-white difference between the two devices was 0.07 mm (95% confidence interval of mean difference: 0.04-0.11 mm). The Pearson correlation coefficient between the two devices was 0.85 (p<0.0001). The 95% limits of agreement between the two devices were -0.38 mm to 0.53 mm.

**Conclusions:**

The Atlas topographer and IOL Master 500 can be used interchangeably with respect to white-to-white distance measurements, as the range of differences is unlikely to affect clinical practice and decision making.

## Abstract (pt)

**Objetivo:**

O objetivo deste estudo é comparar as medições de diâmetro corneano de dois dispositivos normalmente utilizados na prática clínica (IOL Master 500 e Atlas topógrafo corneal) para ver se são permutáveis. O fornecimento de informações sobre a permutabilidade de instrumentos poderia eliminar vários testes desnecessários e, consequentemente, reduzir a carga econômica para o paciente e para a sociedade.

**Métodos:**

Nesta série de casos prospectivos e comparativos, a distância do diâmetro corneano foi medida por examinadores independentes utilizando o Topógrafo Atlas (Carl Zeiss Meditec) e o IOL Master 500 (Carl Zeiss Meditec), em um olho de 184 pacientes. A análise estatística foi realizada utilizando o teste t pareado, a correlação Pearson e a análise Bland-Altman para comparar os métodos de medição.

**Resultados:**

As medições médias da distância do diâmetro corneano com o topógrafo Atlas e o IOL Master 500 foram de 12,20 ± 0,44 mm e 12,12 ± 0,41 mm, respectivamente (p<0,001). A diferença média de WTW entre os dois dispositivos foi de 0,07 mm (intervalo de confiança de 95% da diferença média: 0,04 - 0,11 mm). O coeficiente de correlação Pearson entre os dois dispositivos foi de 0,85, p<0,0001. Os limites de concordância de 95% entre os dois dispositivos foram de -0,38 mm a 0,53 mm.

**Conclusões:**

O Atlas topographer e o IOL Master 500 podem ser utilizados permutavelmente em relação à medição do diâmetro corneano, uma vez que a gama de diferenças encontradas é pouco susceptível de afetar a prática clínica e a tomada de decisões.

## INTRODUCTION

Adequate measurement of the horizontal corneal diameter [white-to-white (WTW) distance] has become increasingly important in ophthalmic practice^(1)^. Newer generations of intraocular lens (IOL) formulas, such as Holladay 2, require accurate measurement of the WTW distance^(2)^. A recent study showed that the actual lens position, among other variables, correlated independently with the WTW distance^(3)^. In addition, the WTW distance can be used to estimate the inner anterior chamber width or ciliary sulcus size to determine the size of an anterior chamber or sulcus-implanted IOL^(4,5)^. Accordingly, the horizontal WTW distance is also used as a parameter in contact lens fitting and it is especially important in scleral lens selection^(1)^.

Several methods (manual and automated) have been described for measuring the horizontal corneal diameter. There is currently no gold standard; however, the reliability and repeatability of automated measurement methods are better than manual methods^(6,7)^. Currently, eye clinics are overcrowded with multiple devices capable of measuring the corneal diameter. Providing information on instrument interchangeability could eliminate unnecessary multiple tests, reducing the economic burden on both the patient and society. Agreement of WTW distance measurements between the IOL Master 500 and Atlas topographer has not been reported, based on a search of the PubMed database. The purpose of this study was to assess the agreement and interchangeability of these two devices in measuring the WTW distance in normal candidates for cataract surgery. The present study has direct clinical relevance, as both the Atlas topographer and the IOL Master are commonly used devices for determining WTW distances.

## METHODS

In this prospective comparative study, 183 patients between the ages of 11 and 90 years who were referred to our outpatient clinic were consecutively enrolled. Only one eye per patient was included in the analysis. If both eyes were eligible, the right eye of each patient was included. Exclusion criteria were poor fixation, limbal pathologies, such as pterygium or pannus, and cases where it was not possible to obtain good quality images from the edge of the iris.

Institutional review board/ethics committee approval was obtained, and the tenets of the Declaration of Helsinki were followed in this study. Informed and signed consent for the research was obtained from each subject prior to enrollment.

The same experienced examiner performed all measurements with two devices. The measurements were performed sequentially with the IOL Master 500 (Carl Zeiss, Meditec, Jena, Germany) and the Atlas topographer (Carl Zeiss, Meditec, Jena, Germany) according to the manufacturer’s instructions. For each device, measurements were repeated as needed until an image of acceptable quality was obtained. For the IOL Master, after taking the image, the operator checked whether the software had correctly detected the edge of the iris. If the circle segments drawn in the image did not correctly define the iris, the result was discarded. The calibration was rechecked before each measurement.

Statistical analysis was performed using JMP statistical software (version 14.0; SAS Institute, Inc, Cary, NC). A paired *t*-test was used to compare the differences in means between the two instruments to determine if there was a systematic shift in the differences between the instruments. A Bland-Altman plot was used to graphically represent the agreement between the two instruments. The 95% limits of agreement (LoAs) were calculated as the mean difference between the tests ± 1.96 standard deviations of the difference between the two tests. The LoAs were interpreted as the range in which 95% of the differences between the two instruments would fall^(8)^. The limits of maximum acceptable differences [also known as limits of clinically acceptable differences (CAD)] were defined *a priori* based on biologically and analytically relevant criteria as ± 0.5 mm. The Pearson correlation coefficient was calculated to assess the correlation between the two measurements for each subject. A p value of <0.05 was considered statistically significant.

## RESULTS

**Figure 1:**
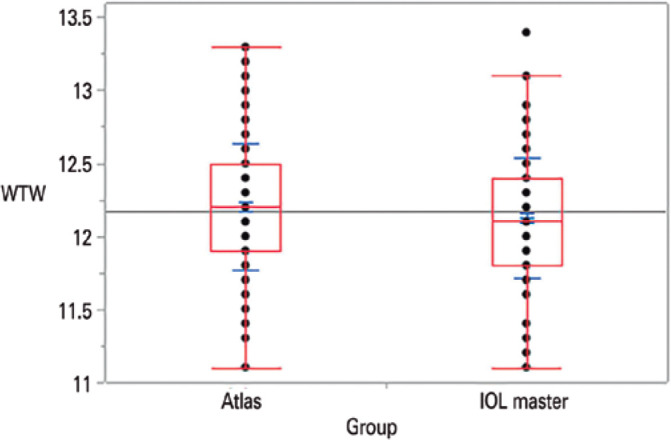
Box-plot of white-to-white distance measurements obtained with the Atlas topographer and IOL Master.

**Figure 2:**
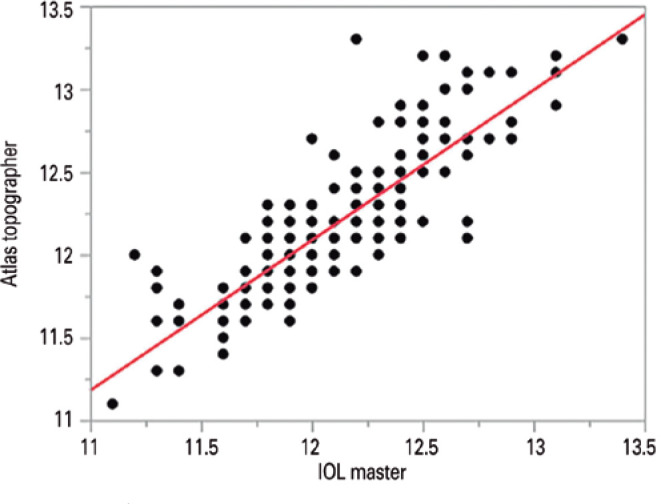
Correlation between white-to-white distance measurements obtained with the Atlas topographer and IOL Master.

**Figure 3:**
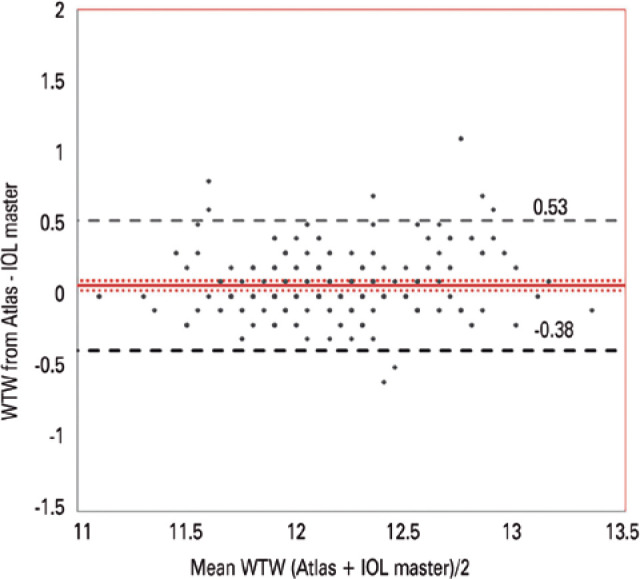
Bland-Altman Plot of white-to-white distance values measured with the Atlas topographer and IOL Master. Limits of agreement for the Atlas topographer and IOL Master fell between -0.38 to + 0.53 mm with a relatively uniform distribution. Red line = bias; red dot lines = 95% confidence interval of the bias; dashed lines = 95% limits of agreement.

This study enrolled 63 men and 120 women. The mean age was 62.8 ± 16.63 years (range, 11-90 years). The mean WTW distance readings were 12.20 ± 0.44 mm (range, 11.1-13.3 mm) as obtained with the Atlas topographer and 12.12 ± 0.41 mm (range, 11.1-13.4 mm) as obtained with the IOL Master (Figure 1). The mean difference between the device measurements was 0.07 ± 0.23 mm (95% confidence interval, 0.04-0.11; p<0.0001). In 25.7% of the eyes, the measurements were exactly the same and only 7.7% of eyes had diffe­rences of more than 0.5 mm. The Atlas topographer measured the WTW distance greater than the IOL Master in 45.9% of the cases and in 28.4% it was in the opposite direction. The measurements were highly correlated (Pearson correlation coefficient = 0.85; 95% confidence interval, 0.80-0.89; p<0.0001; Figure 2). As assessed with a Bland-Altman plot, the variation of the differences between the two devices was reasonably constant over the range of the measurements. According to the Bland-Altman plot, there was not a significant systematic bias, because the line of equality was within the confidence interval of the mean difference. The 95% LoAs were -0.38 to 0.53 mm (Figure 3).

This study enrolled 63 men and 120 women; mean age, 62.8 ± 16.63 years (range, 11-90 years). The mean WTW distance measurements were 12.20 ± 0.44 mm (range, 11.1-13.3 mm) as determined with the Atlas topographer and 12.12 ± 0.41 mm (range, 11.1-13.4 mm) as determined with the IOL Master (Figure 1). The mean difference between the measurements of the devices was 0.07 ± 0.23 mm (95% confidence interval, 0.04-0.11; p<0.0001). In 25.7% of the eyes, the measurements were exactly the same, and only 7.7% of the eyes had differences of more than 0.5 mm. The Atlas topographer measured a larger WTW distance than the IOL Master in 45.9% of cases and in 28.4% it was the oppo­site situation (0.4%). The measurements were highly correlated (Pearson correlation coefficient = 0.85; 95% confidence interval, 0.80-0.89; p<0.0001; Figure 2). As can be seen from the Bland-Altman plot, the variation in differences between the two instruments was constant over the range of measurements. According to the Bland-Altman plot, there was no significant systematic bias, because the line of equality was within the confidence interval of the mean difference. The 95% LoAs ranged from -0.38 to 0.53 mm (Figure 3).

## DISCUSSION

The importance of accurately measuring the WTW distance is well acknowledged. In the past, the WTW distance was mainly used for the diagnosis of congenital glaucoma and micro- or megalocornea; however, recently it has also become relevant in the planning of cataract surgery, as the newer generation formulas, such as Holladay 2, Hill-RBF, Olsen and Barrett Universal II, recommend use of the WTW distance as an input variable^(1,2)^. It is also imperative for selecting the correct size of an anterior chamber or sulcus-implanted IOL^(4,5)^. However, despite many instruments that can measure it, a thorough review has shown that the accuracy and interchangeability of these instruments remains questionable and neither can be used as the gold standard^(7)^. The results of this study showed that the IOL Master and Atlas topographer may be interchangeable with respect to WTW distance measurements.

The present study has direct clinical relevance, as both the Atlas topographer and IOL Master are commonly used devices to determine WTW distances. Although the mean difference (bias) between the two devices was statistically significant, it was clinically irrelevant (<0.5 mm). Meanwhile, the measurements were highly correlated and there was no significant systematic bias based on the Bland-Altman plot, with differences scattered around the bias and with no obvious pattern.

To decide whether two measurement systems agree sufficiently to be used interchangeably, one must compare the LoAs to the CAD; that is, the maximum allowable difference between two measurements^(8)^. In this study, the LoAs (-0.4 to 0.5 mm) were within the CAD (-0.5 to 0.5 mm), so it is likely that the measurements of the two systems will not differ by more than the allowable amount rarely. We concluded that the two measurement systems agree sufficiently.

The accuracy of limbus recognition by the computer software for the automated methods depends on the quality of the anterior segment images^(9)^. The Atlas Corneal Topography System (Carl Zeiss Meditec, Jena, Germany) has a patented alignment system, and its ability to analyze multiple images during the alignment phase means it will automatically select the highest quality image. It uses a Placido disc-based data acquisition system designed for rapid, quantitative photokeratoscopy to capture the anterior segment’s topographic features. The corneal diameter is automatically calculated by the computer and the examiner does not need to validate the image to see if the limbus was correctly marked for the WTW distance measurement. The IOL Master (Carl Zeiss Meditec, Jena, Germany) measures the WTW distance based on the digital “photographic” image it captures. This device then digitally locates the limbus based on a sudden change in contrast from light sclera to dark cornea. After the image is captured, the operator checks to see if the software has correctly identified the edge of the iris. If the circle segments drawn in the image do not correctly define the iris, the result must be discarded.

Measurements of corneal diameters with the IOL Master have already been studied, and acceptable accu­racy and repeatability have been reported^(7,10-16)^. The IOL Master 500 uses the principle of partial coherence interferometry to obtain measurements of the axial length (AL) with high precision. The IOL Master 700 was the first swept source optical coherence tomography (OCT) used for biometry and it was recently introduced. Although the IOL Master 500 and 700 differ in the technology used to obtain AL, the WTW acquisition method is not difference, which is measured with a light-emitting diode light source according to the iris configuration^(17)^. Previous studies examining the agreement between the IOL Master and Orbscan IIz showed that the mean WTW distance measurements were approximately 0.24-0.32 mm higher with the IOL Master^(7,11)^. Accordingly, a statistically significant lower WTW distance was also found with the Lenstar (Haag Streit, USA) and with the Scheimpflug/Placido topography compared with the IOL Master^(15,18)^. However, in another study, the IOL Master overestimated WTW distance measurements by up to 0.78 mm compared with the Pentacam HR, and the authors concluded that these two devices should not be used interchangeably for this purpose. Agreement was slightly weaker in eyes with WTW distances of 11.50 mm or less compared with eyes with WTW distances greater than 11.50 mm^(19)^. In the present study, the IOL Master provided lower values than the Atlas topographer, but the difference was not considered clinically relevant. Because the different studies included different cohorts of patients and used different methods, comparisons between these studies should be made with caution.

This study included a sufficient sample size and included only one eye of each patient in the analysis, both of which improved the validity of the statistical analysis. However, the results of this study are limited to the measurement range studied (11.1-13.4 mm) and should not be extrapolated to eyes with smaller or larger WTW distance measurements or corneas with pathologic changes (such as pannus or other peripheral corneal changes). An even greater bias is expected with these conditions. Patients in this study were recruited from a continuous cohort. Because the inclusion criteria were not stringent, our study population represented patients who were being evaluated in routine clinical practice.

A limitation of this study was that we cannot confirm which device measures WTW distance more accurately, as there is no gold standard for WTW measurement.

In conclusion, the Atlas topographer and IOL Master can be used interchangeably for WTW distance measurements, as the differences found are unlikely to affect clinical practice and decision making, can eliminate multiple unnecessary tests, save time, and consequently reduce the economic burden on the patient and society. However, because only relatively normal corneas of candidates for refractive or cataract surgery were measured in this study, the relevance of these results to corneas with pathologic changes remains unknown.
